# The RosettaCon 2012 Special Collection: Code Writ on Water, Documentation Writ in Stone

**DOI:** 10.1371/journal.pone.0073775

**Published:** 2013-09-26

**Authors:** Ingemar André, Jacob Corn

**Affiliations:** 1 Department of Biochemistry and Structural Biology, Lund University, Lund, Sweden; 2 Department of Early Discovery Biochemistry, Genentech Inc., South San Francisco, California, United States of America; University of South Florida College of Medicine, United States of America

Rosetta is a powerful software suite for the modeling and design of macromolecules [1]. Originally written within the laboratory of David Baker, the Rosetta developers community (RosettaCommons, https://www.rosettacommons.org/) has expanded to encompass hundreds of developers across tens of institutions. The Rosetta community, from software developers to academic and industry users, meets yearly to discuss exciting new advances, with 2012 marking the tenth anniversary RosettaCon. This 2012 Special Collection captures a selection of the scientific advancements in the two years since the last RosettaCon Special Collection [2].

## Reproducibility and Computational Biology

In addition to highlighting exciting science, one of the primary goals of the RosettaCon Special Collections is to address issues of reproducibility in computational biology [3], [4]. At first glance, “dry” computational biology seems inherently more reproducible than its “wet” experimental counterpart. All input to a computational experiment is precisely known and controlled, and the output is generated by a well-defined algorithm that is also under the programmer's control. In practice, reproducibility often suffers when the formatting requirements of a journal meet the massive datasets and complex workflows of modern computational biology.

Computational methods often synthesize a wide range of techniques to reach an interesting result, with multi-layered tasks that are difficult if not impossible to reproduce with a single command line argument. Yet documentation in the Methods section of a manuscript is rarely complete enough for outside experts to replicate these complex experiments, since the details of a protocol often mean the difference between success and failure. Monte Carlo algorithms can be particularly susceptible to these traps, since input data is often pre-processed, simulation output is stochastically generated, and the output is often significantly post-processed to synthesize a meaningful result. Without an accompanying test case, constant random number seed, and thorough description of the sampling necessary to obtain reasonable output, individuals attempting to replicate data may never be able to determine the root cause of “funny” results. Even in cases where core protocols are laboriously described, specialized pre- or post-processing scripts and programs written by former lab members further complicate matters and may even prevent replication within the originating lab.

Several causes may underly poor documentation and code distribution, including a reward system built upon high-profile papers as opposed to robust frameworks. But at the end of the day, the whole fields suffers as groups are forced to re-learn lessons obscured by time and poor documentation. Some projects are notable exceptions, such as bioperl/python/java and bioconductor [5]–[8], which freely distribute their source code under open source licenses and incorporate extensive documentation and tutorials. Not coincidentally, these projects enjoy widespread adoption and use, with tens to hundreds of thousands of downloads per year (http://www.bioperl.org/wiki/Getting_BioPerl, http://biopython.org/wiki/Download, http://www.bioconductor.org/packages/stats/).

## Overview of Rosetta and the PLoS Collection

The Rosetta macromolecular modeling suite also enjoys widespread use, yet in the past has suffered from incomplete documentation, partially due to its extremely active development. Rosetta was originally developed for ab-initio protein structure prediction [9] but has evolved into a multi-purpose program that includes methods for template based modeling [10], protein-protein [11], [12] and protein-DNA design [13], enzyme design [14], [15], protein-protein [16] and protein-ligand docking [17], structure inference from limited experimental data [18], RNA structure prediction [19] and design, and peptidomimetic design [20].

Rosetta's rapid growth is fueled by the RosettaCommons, which is a non-profit entity that coordinates the development of the program and handles academic and commercial licensing. RosettaCommons (http://www.rosettacommons.org) is a collaboration between more than 15 research groups involved in the development of the Rosetta code base. The revenue generated by commercial licenses funds infrastructure for validation of code developments, users support, and developer meetings. The philosophy behind RosettaCommons is further described in the overview paper presenting the 2010 RosettaCon meeting [2].

In addition to addressing scientific problems via the Rosetta macromolecular modeling suite, the papers presented in this special collection tackle problems of reproducibility and documentation head-on. Publication of a paper in the collection is conditioned on the submission of an archive containing links to the exact version of the code used in the paper, all input data, links to external tools, and an example script to illustrate the use of the code to carry out the protocol described in the paper. In addition, the paper is required to contain a detailed procedural description in the methods section. This “protocol capture” approach has also inspired a set of guidelines for how to present Rosetta computational workflows outside the PLOS collection. Importantly, the procedural description is used to audit each article, such that all protocols and documentation have been independently followed and verified to be complete by individuals outside the authors' laboratory. The large amount of testing data involved in this documentation is available via the RosettaCommons website (http://www.rosettacommons.org).

Naturally, while exhaustive documentation is necessary to recreate or modify a protocol, some users simply wish to try an established workflow on their favorite system, without spending large amounts of time deeply understanding the underlying theory or replicating test cases. However, the usage of many computational methods, including Rosetta, still requires considerable computational fluency and access to large computational resources, prohibiting wider use. This year's RosettaCon special collection addresses this need with the inclusion of ROSIE (Rosetta Online Server that Includes Everyone) [21], a general framework for the rapid development of public Rosetta web servers. Lowering the barrier to entry for the use of Rosetta protocols will hopefully democratize their use, such that the power of Rosetta becomes more accessible to a general audience.

## Summary of papers

This special issue includes articles that describe a wide variety of aspects relating to the application of Rosetta in structure prediction and design. The articles can be divided into three categories: increasing the usability of Rosetta, improvements to current structure prediction methods, and completely new Rosetta procedures and applications. Each article is supplemented with full a “protocol capture,” including documentation, test data, and processing scripts that have been peer reviewed by individuals outside the developers' research group. In a few cases the protocol capture is supplanted by a ROSIE web server interface to the application.

### Lowering barriers to using Rosetta

Two articles in this special issue describe advancements that significantly lower the barriers for non-experts to use complex Rosetta applications. Lyskov et al. [21] introduce ROSIE (Rosetta Online Server that Includes Everyone); a framework for the serverification of Rosetta protocols. The ROSIE workflow allows Rosetta developers to rapidly convert Rosetta applications into web servers, all of which run on common hardware resources. This framework allows for the development of fully functional web servers for Rosetta applications within a few weeks. In a time scale of a few months nine servers based on the ROSIE framework have been launched, including two of the new applications described in this special issue [22], [23].

Another means for lowering the barriers for non-experts is to provide a graphical user interface (GUI) to Rosetta. Adolf-Bryfogle and Dunbrack [24] describe the development of a GUI called the PyRosetta Toolkit, which allows users to to create and run common Rosetta molecular modeling and protein design tasks as well as analyze the results of Rosetta calculations. New applications can rapidly be modified to take advantage of the PyRosetta Toolkit.

### Improvements to current structure prediction methods

Several articles describe improvements in Rosetta's structure prediction and design methodology. Drew et al [23] develop a framework to represent “nancanonical” peptidomimetic backbones in Rosetta, allowing the modeling and design of molecules such as peptoids and oligooxopiperazines. Notably, peptidomimetic design has already been incorporated into a ROSIE server. Alexander et al. [25] also explore the addition of new chemistries to Rosetta via improvements to RosettaEPR, a framework for using Electron Paramagnetic Resonance data to improve structure prediction. The new version of RosettaEPR includes a new rotamer library for a common spin label and more accurate reproduction of experimentally determined distance distributions.

Due to the astronomical size of protein conformation space, sampling is a long-standing bottleneck for Rosetta. Stein and Kortemme [26] find that significant improvements in loop conformational sampling can be achieved by combining several sampling strategies in the context of Rosetta. This strategy extends the KIC method [27] to yield even more accurate predictions of local conformations of proteins. Zhang and Lange [28] also tackle sampling, finding that a replica exchange approach greatly improves conformational sampling during the low resolution stage of RosettaDock. Khar et al. [29] have recently developed a ray-casting method (DARC) for small molecule docking and now demonstrate that its speed can be increased 25-fold via GPU-based computing, thereby enabling virtual screening of large compound libraries.

### New Rosetta procedures and applications

New computational procedures and applications often debut at RosettaCon, and this issue contains several articles describing new Rosetta methodology. Lemmon and Meiler [30] introduce two methods for dealing with the challenging problem of performing small ligand docking with explicit interface water. Dong Nguyen et al. [31] provide a method for ligand docking into homology models of G-protein coupled receptors and present extensive benchmarking results. Although Rosetta protein design has recently achieved some landmark successes [12], [13], [15], [32], the preparation of template “scaffold” proteins is non-trivial. Nivon et al [33]. describe a procedure to optimally pre-refine scaffold proteins prior to the computational design of functional sites. Computational design is also discussed by Der et al. [22], who explore two methods of automatically supercharging of protein surfaces to increase solubility. The authors experimentally test the performance of each method and have already made the supercharging protocol available as a ROSIE web server. Finally, Kahraman et al. [34] introduce protocols to drive both Rosetta de novo modeling and protein docking via the incorporation of experimental cross-linking data, as well as describe a structure-based crosslink database.

## Conclusion

The Rosetta community has rapidly grown from a single lab to hundreds of people across many institutions, all contributing to (as of April, 2013) more than 1 million lines of code. As Rosetta expands in both users and developers, we must continually strive to keep the software readily available, transparent, and usable. This includes behind-the-scenes efforts, such as automated testing servers to ensure code robustness, as well as public outreach, such as help/announcement forums (https://www.rosettacommons.org/forum) and workshops (http://structbio.vanderbilt.edu/comp/workshops/rosetta_13/). The RosettaCon Special Collections and their associated protocol captures offer an accessible window into the fast-moving world of Rosetta development. We look forward to future Rosetta improvements to increase the availability of new Rosetta functionality, such as greatly accelerated release cycles, and hope that efforts such as the Special Collections ensure that bleeding-edge protocols are as usable as more established workflows.
